# CircFISH: A Novel Method for the Simultaneous Imaging of Linear and Circular RNAs

**DOI:** 10.3390/cancers14020428

**Published:** 2022-01-15

**Authors:** Aakash Koppula, Ahmed Abdelgawad, Jlenia Guarnerio, Mona Batish, Vijay Parashar

**Affiliations:** 1 Department of Biological Sciences, University of Delaware, Newark, DE 19716, USA; aakash@udel.edu (A.K.); ahmedg@udel.edu (A.A.); 2 Department of Medical and Molecular Sciences, University of Delaware, Newark, DE 19716, USA; 3 Samuel Oschin Comprehensive Cancer Institute, Division of Cancer Biology and Therapeutics, Cedars-Sinai Medical Center, Los Angeles, CA 90048, USA; Jlenia.Guarnerio@cshs.org

**Keywords:** single-molecule RNA imaging, circular RNAs, RNase R, nucleic acid probes, non-coding RNAs, fixed cells, FFPE, frozen tissues, multiplex RNA imaging, immunofluorescence, subcellular RNA localization

## Abstract

**Simple Summary:**

Circular RNAs are a recently appreciated class of regulatory non-coding RNAs. Although a number of high-resolution methods have been developed for the imaging of RNAs in cells and tissues, there is no reliable method for the simultaneous imaging of circular RNAs distinctly from their linear counterparts. Here, we report circFISH as a simple and single-molecule resolution method that will simultaneously image linear and circular RNAs in fixed cells and tissues. We demonstrate that multiple circular RNAs can be imaged using circFISH. We also show the ability of circFISH to work seamlessly with protein and other organelle imaging. We optimized the method to work across sample types, making it a versatile tool for the functional characterization of circular RNAs.

**Abstract:**

Circular RNAs (circRNAs) are regulatory RNAs which have recently been shown to have clinical significance in several diseases, including, but not limited to, various cancers, neurological diseases and cardiovascular diseases. The function of such regulatory RNAs is largely dependent on their subcellular localization. Several circRNAs have been shown to conduct antagonistic roles compared to the products of the linear isoforms, and thus need to be characterized distinctly from the linear RNAs. However, conventional fluorescent in situ hybridization (FISH) techniques cannot be employed directly to distinguish the signals from linear and circular isoforms because most circRNAs share the same sequence with the linear RNAs. In order to address this unmet need, we adapted the well-established method of single-molecule FISH by designing two sets of probes to differentiate the linear and circular RNA isoforms by virtue of signal colocalization. We call this method ‘circular fluorescent in situ hybridization’ (circFISH). Linear and circular RNAs were successfully visualized and quantified at a single-molecule resolution in fixed cells. RNase R treatment during the circFISH reduced the levels of linear RNAs while the circRNA levels remain unaltered. Furthermore, cells with shRNAs specific to circRNA showed the loss of circRNA levels, whereas the linear RNA levels were unaffected. The optimization of the in-situ RNase R treatment allowed the multiplexing of circFISH to combine it with organelle staining. CircFISH was found to be compatible with multiple sample types, including cultured cells and fresh-frozen and formalin-fixed tissue sections. Thus, we present circFISH as a versatile method for the simultaneous visualization and quantification of the distribution and localization of linear and circular RNA in fixed cells and tissue samples.

## 1. Introduction

A variety of regulatory RNAs have been reported to play important roles at different stages of gene expression regulation [1,2,3,4]. The most recent additions to this growing repertoire of regulatory RNAs are circular RNAs (circRNAs) [5,6,7]. As the name suggests, circRNAs exist as closed-loop structures with no open 5′ or 3′ ends, and constitute about 1% of total RNAs [8]. CircRNAs are generated by a process known as back-splicing [9]. In canonical splicing, exons are spliced and brought together in a sequential fashion to remove introns and produce linear transcripts. However, in the case of back-splicing, a downstream 5′ splice site is brought into close proximity to an upstream 3′ splice site, either through the complementary binding of inverted intronic repeats (e.g., Alu repeats), or through the activity of RNA binding proteins, both of which result in a looped product known as circRNA [10,11]. Back-splicing competes with the canonical splicing, and thus a given gene transcript can form a different number of linear and circular isoforms. The level of expression of each isoform is believed to be regulated, and perturbations in this balance are believed to affect cellular pathology [12]. Although circRNAs were first reported in 1970s, they were often believed to be errors of splicing until the last decade, when their roles in various physiological processes became increasingly appreciated [13]. CircRNAs have now been established as key regulatory RNAs that are conserved across species and have tissue-specific expression, with their levels in the cells regulating various diseases [14]. Due to their closed-end structure, circRNAs are resistant to exonucleases, and thus have about a five times longer half-life than linear isoforms [15]. These circRNAs have also been reported to be enriched in extracellular vesicles, highlighting their emerging role as promising biomarkers in various pathologies like neurological disorders, cardiac diseases, and many cancers [10,16]. Although thousands of circRNAs have been identified, only a handful have been functionally characterized [10]. The most well-studied function of circRNAs which has been characterized so far is their role as sponges for microRNAs and RNA binding proteins [14,17]. Recent studies have identified distinct and even antagonistic functions of circRNAs compared to the protein products of the linear isoforms originating from the same genetic sequence [18]. The circRNAs could be composed of single or multiple exons (exonic circRNAs), introns alone (intronic circRNAs), or even a combination of both exons and introns (exonic-intronic circRNAs). The exonic circRNAs are the most abundant, and are reported to have the most clinical relevance [8,15,19,20,21].

Most studies on circRNA characterization rely on the identification of a unique back-splice junction (BSJ) that is formed due to their closed circular structure. A pair of divergent primers are often used in Polymerase Chain Reactions (PCRs) to selectively amplify circRNAs [22]. The PCR products are then Sanger sequenced to confirm the presence of the BSJ [10]. Another method of validation is by treating the total cellular RNA with an exonuclease, RNase R, which degrades the linear but not circRNA forms due to the absence of open ends in circRNAs [23]. RNase R treatment is also used to enrich circRNAs for the purpose of in RNA sequencing or microarray experiments aiming to identify circRNAs [24]. A number of computational tools have been developed based on these datasets to predict the presence and functional interactions of different circRNAs (reviewed in [25]). All of these analyses only provide information on the presence of a given circRNA in a population of cells, and they often lack details about the spatial and contextual information of circRNAs. This information is critical for regulatory RNAs, as their localization largely dictates their function in the cell. Furthermore, because it is well appreciated that transcription is stochastic, studies using a population of cells pooled together lose the information regarding the cellular heterogeneity in circRNAs’ expression in individual cells. Most of the pertinent insights regarding the transcription and functional characterization of various RNA species were obtained using methods of single-molecule resolution imaging [26]. High-resolution imaging also provides insights into the heterogeneous expression in clinical samples, especially in solid tumors where cells at different stages of tumorigenesis can coexist in the tumor microenvironment. Therefore, it is very important to develop novel techniques to image the distribution and associations of circRNAs at a high resolution [27].

Although several techniques have been developed to achieve the high- and super-resolution imaging of various steps in the RNA processing, the adaptation of these methods for the imaging of circRNAs is still in its early stages. The fact that circRNAs share their sequence with their linear counterparts confounds the direct applicability of these techniques to differentiate circular and the linear isoforms.

Most of the currently available circRNA imaging methods rely on the targeting of the unique BSJ in the circRNAs. The traditional RNA fluorescence in situ hybridization (RNA FISH) targeting of the BSJ has been used to image circRNAs [28]. For example, it is used in BaseScope, which is a commercial assay that uses a series of in situ probes to selectively target BSJs in circRNAs [29]. Recently, linear DNA nanostructures were developed to image circRNAs [30]. These and other similar imaging modalities were recently reviewed extensively by Bejugam et al. [27]. This review also highlighted the unmet need for sensitive and reliable methods for circRNA imaging. Although the BSJ targeted by these methods is a specific signature sequence, it is only present in one copy per circRNA. The presence of sequences similar to BSJ therefore reduces the specificity of these approaches. Finally, the multiplexing of these assays is restricted due to the availability of only a limited number of enzyme substrate pairs that are used for signal development in the majority of these methods.

The single-molecule resolution of single-molecule FISH (smFISH) has been used to image intronic and intergenic circRNAs by designing probes which are specific for circRNA sequences [27,31]. However, smFISH cannot be used directly for exonic circRNAs imaging, which constitute more than 80% of clinically relevant circRNAs [32]. We have previously adapted smFISH as Fusion FISH for the imaging of the gene fusion transcripts [33]. The sensitivity and specificity of smFISH has been demonstrated in various systems, as well as in diverse biological contexts by multiple independent laboratories [26]. 

The method of smFISH utilizes a set of short (20 nucleotides each) single-labeled probes that tile along the length of the target RNA. The binding of most of the probes together in proximity provides a diffraction-limited spot in the fluorescence microscope where each spot generated represents a single RNA molecule [34]. The signal is highly specific, as any off-target binding of a few probes does not yield a signal above the general background, thus providing a very high signal-to-noise ratio. The signal obtained can be quantified with image processing software with a sensitivity comparable to quantitative real-time PCR [35]. In this study, we demonstrate a successful adaptation of smFISH for the sensitive and specific imaging of circRNAs distinctly from their linear counterpart for a given gene. We call this method circFISH.

## 2. Materials and Methods

### 2.1. Cell Lines and Cloning

DLD-1, HS-5 and HEK-293T (CRL-3216, ATCC) cell lines were cultured in DMEM (Millipore Sigma, St. Louis, MO, USA, D6429) supplemented with 10 % FBS (Millipore Sigma, St. Louis, MO, USA, F2442) and 1% penicillin/streptomycin (Millipore Sigma, St. Louis, MO, USA, P4333). The DLD-1 and HS-5 cell lines were a generous gift from the Leung lab ( Johns Hopkins, MD, USA) and Guarnerio labs (Cedar Sinai Medical Center, CA, USA), respectively. The A549 cells were cultured in F-12K Medium (Kaighn’s Modification of Ham’s F-12 Medium) (Gibco, Grand Island, NY, USA, 21127-022) supplemented with 10% FBS and 1% penicillin/streptomycin. The A549 cell line was a gift from Kmeic lab.

The shRNA targeting the BSJ of circCSNK1G3 was cloned into the pLKO.1-TRC cloning vector (Addgene, Watertown, MA, USA, Plasmid # 10878) and transfected into HEK-293T cells for virus production. A scrambled shRNA sequence was cloned into the pLKO.1-TRC cloning vector, and was used as a negative control. DLD-1 cells were transduced with a virus, and stable cell lines were selected using 1.25 ug/mL puromycin (Gibco, Waltham, MA, USA, A11138-03).

All of the cells were cultured at 37 °C and 5% CO_2_. For the imaging experiments, all of the cells were grown on 0.1% gelatin (Bio-Rad, Hercules, CA, USA, 170-6537) coated coverslips in 100 mm dishes until 70–80% confluency before fixing in 4% formaldehyde (Millipore Sigma, St. Louis, MO, USA, F8775) and permeabilizing in 70% ethanol for hybridization.

### 2.2. Probe Synthesis and Purification

The sequences of linear and circular isoforms were obtained from Ensembl (https://useast.ensembl.org/index.html, last accessed date: 1 July 2020) and CircInteractome (https://circinteractome.nia.nih.gov/api/v2/circsearch?circular_rna_query=hsa_circ_0000615, last accessed date: 1 July 2020). In order to generate the PC and PL probes, sets of linear oligonucleotide probes, each 20 nucleotides in length, were designed to be complementary to specific regions of the target RNA molecules with a 3′ amino group modification using the Stellaris Probe Designer program from Biosearch Technologies, Novato, CA, USA. For *ZNF609* and *ZBTB7a*, a set of 35 probes were each designed for the PC and PL probe sets. For *CSNK1G3*, a set of 33 probes was used in the PL probe set and 24 probes were used in the PC probe set. All of the probes had a GC content within 35 to 55% in order to ensure optimal binding under hybridization conditions. The probes were pooled in equimolar concentrations, and were conjugated with Texas Red (TR) (Invitrogen, Waltham, MA, USA, T6134) or Cy5 (GE Healthcare, Chicago, IL, USA, PA25001) fluorophores, and then purified by high-pressure liquid chromatography (HPLC), as previously described [35]. A list of all of the probe sequences is provided in Supplementary Table S1.

### 2.3. In-Situ CircFISH Hybridization

Coverslips with fixed adherent cells were washed with 2X saline sodium citrate solution (Ambion, Austin, TX, USA, AM9763) containing 20% formamide (Ambion, Austin, TX, USA, AM9342/44) and 2 mM ribonucleoside–vanadyl complex (New England Biolabs, Ipswich, MA, USA, S1402S); then, the cells were hybridized with probe sets in 50 μL hybridization buffer (containing 10% (*wt*/*vol*) dextran sulfate (Sigma, St. Louis, MO, USA, D8906), 1 μg/μL yeast tRNA (Invitrogen, Waltham, MA, USA, 15401-029), 2 mM ribonucleoside–vanadyl complex to inhibit ribonucleases, 0.02% (*wt*/*vol*) ribonuclease-free bovine serum albumin (Ambion, Austin, TX, USA, AM2618), 20% (*vol/vol*) formamide dissolved in 2X saline sodium citrate solution, and 25 ng/μL of each probe set. The hybridization was carried out overnight at 37 °C in a moist chamber. On the next day, the coverslips were washed four times with 20% formamide in 2X saline sodium citrate solution, stained with DAPI (Sigma, St. Louis, MO, USA, D9542), and mounted in mounting media as described previously [35].

### 2.4. In-Situ RNase R Treatment

Coverslips with fixed adherent cells were washed with 2X saline sodium citrate solution containing 20% formamide and 2 mM ribonucleoside–vanadyl complex; then, the cells were subjected to RNase R treatment at 37 °C for various time periods using 2U RNase R (Epicenter, Madison, WI, USA, RNR07250) per 1ug RNA. The coverslips were later washed with 2X saline sodium citrate solution containing 20% formamide and 2 mM ribonucleoside–vanadyl complex four times, and then in situ hybridization was carried out as described above.

### 2.5. Simultaneous CircFISH and Immunofluorescence

Immunofluorescence staining was coupled with circFISH for the visualization of the colocalization of linear and circular RNAs with the protein targets. The coverslips containing fixed cells were washed with 2X saline sodium citrate solution containing 20% formamide and 2 mM ribonucleoside–vanadyl complex, then hybridized with probe sets in 50 μL hybridization buffer and incubated overnight at 37 °C in a moist chamber. On the next day, the coverslips were washed with 2X saline sodium citrate solution three times for 15 min, and then incubated with 3 mg/mL bovine serum albumin (BSA) (Ambion, Austin, TX, USA, AM2618) in 2X saline sodium citrate solution for 1 h at RT. The coverslips were then incubated with primary antibodies for AGO2 (Sigma, St. Louis, MO, USA, SAB4200085) and KDEL (Thermo Fischer, Waltham, MA, USA, PA1-013-A488) for 2 h at 37 °C, and then washed three times with 2X saline sodium citrate solution, 10 min each, followed by incubation with secondary antibody (EMD Millipore, Burlington, MA, USA, AP183F) for 1 h at RT in the case of AGO2. The coverslips were then processed for imaging as described above.

### 2.6. Fluorescence Imaging and Analysis

The images were captured with a 100× oil objective using a Nikon TiE Inverted epi fluorescence microscope equipped with a pixis 1024 b camera (Princeton Instruments, Princeton, NJ, USA). The images were obtained using Metamorph imaging software, version 7.8.13.0 (Molecular Devices, MA, USA). Z-stack images were captured for each fluorescent wavelength using 2-s exposures, for a total of 16 stacks, 0.2 mm apart. The compiled z-stack images were analyzed using in-house designed algorithm with MATLAB software (MathWorks, Natick, MA, USA) that identifies signals in each image and determines their three-dimensional coordinates, then identifies spots that have a counterpart within a 250 nm distance in the other channel. Spots meeting this criterion are classified as co-localized. Each imaging experiment was performed in triplicate, and at least 100 cells were counted to obtain the average RNA counts. The error bars indicate a 95% confidence interval. The *p*-values were obtained using Student’s *t*-test.

### 2.7. qRT-PCR

The total RNA was extracted from cells lysed in Trizol (Sigma, St. Louis, MO, USA, T9424) using the phenol-chloroform method, following the manufacturer’s protocol. The RNA purity and concentrations were measured using Nanodrop (Thermo Scientific, Waltham, MA, USA, ND-2000). Equal concentrations of RNA from different cells were used for cDNA synthesis using iScript Reverse Transcription Supermix (Bio-RAD, Hercules, CA, USA, 1708841), and gene expression was analyzed using iTaq Universal SYBR Green Supermix (Bio-RAD, Hercules, CA, USA, 1725124) according to the manufacturer’s protocol. All of the experiments were performed in triplicate. The *p*-values were obtained using Student’s *t*-test. All of the primers used are provided in Supplementary Table S1.

### 2.8. Frozen and Fixed Tissue Samples

The mouse melanoma sections were obtained from Dr. Guarnerio’s lab (Cedar Sinai Medical Center, CA, USA). Briefly, the sections were generated from B16 melanoma cells grown in syngeneic C57BL6 mice in Guarnerio’s lab. The tumor was isolated and fresh frozen, and was then sectioned into 10 μm slices, or it was embedded into formalin and then sectioned into thin slices. The human frozen tissue array was purchased from Amsbio (Abingdon, UK, T6234433), with 8 human tissues fixed on a slide. All of the sections were processed for circFISH using previously optimized protocols for various sample types [35].

## 3. Results

### 3.1. Principle of CircFISH

**Figure 1:**
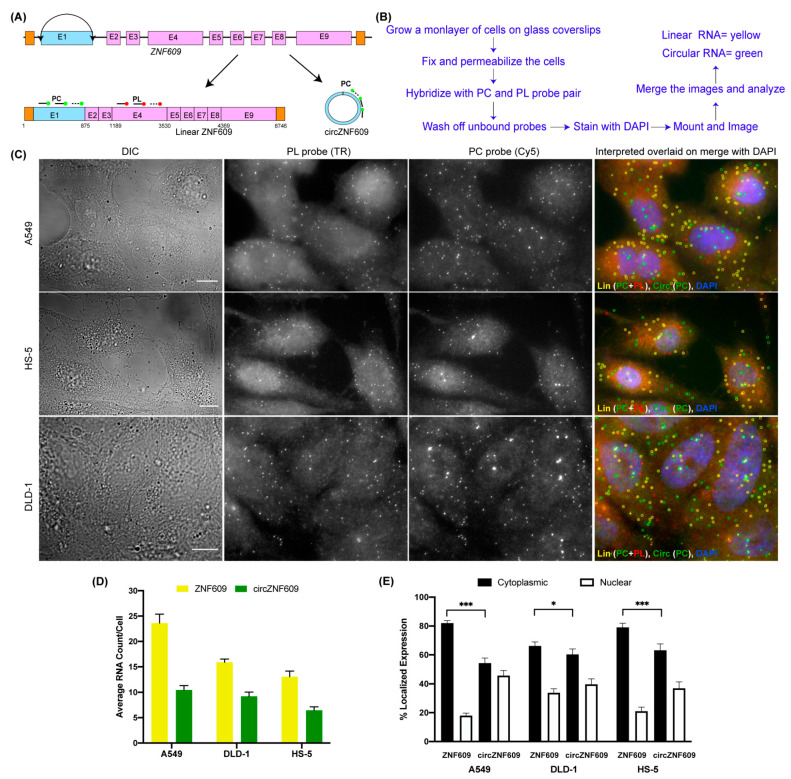
Visualizing circZNF609 using CircFISH. (**A**) Schematic of circFISH for the simultaneous imaging of linear and circular ZNF609. The binding of the PL and PC probes on the linear and circular isoforms, respectively, of ZNF609 is shown by straight lines ending in filled circles. (**B**) Standard protocol for the circFISH method. (**C**) Representative image panel for circFISH for ZNF609 in A549, HS-5 and DLD-1 cells with the PL and PC probes. From the left: DIC; raw merged z stacks of cells for PL probes labeled with TR; raw merged z stacks of cells for PC probes labeled with Cy5; and a merged image of the two channels with TR spots pseudo-colored red and Cy5 pseudo-colored green, overlaid on DAPI with MATLAB-interpreted spots. Full-length linear, circular and fragmented linear RNA is represented as yellow, green, and red spots, respectively. (**D**) Quantification of ZNF609 and circZNF609 signals after MATLAB analysis. The columns represent the average ZNF609 and circZNF609 RNA molecules per cell, as analyzed in MATLAB across the different cell lines. (**E**) Average relative nuclear and cytoplasmic localization of ZNF609 and circZNF609 across the different cell lines. The error bars indicate the 95% confidence interval for at least 100 cells. * Indicates a significant difference with a *p*-value < 0.05. *** Indicates a significant difference with a *p*-value < 0.001. The scale bar is 5 μm.

The idea for circFISH stems from Fusion FISH, where we use two unique sets of probes each labeled with differently colored fluorophores to image distinct parts of chimeric fused mRNAs [33]. Here, we used a well-studied circRNA ZNF609 (zinc finger protein 609) to demonstrate the principle of circFISH [36]. The gene *ZNF609* comprises nine exons; the first exon (some reports call it exon 2 due to the short untranslated region upfront of this exon [36]) undergoes back-splicing and forms a circRNA of 875 nt in size (Figure 1A). Here, we designed two different probe sets, where the first probe set—named probe circular (PC)—specifically binds exon 1, which is a part of the circRNA. Each probe in this set was labeled with Cy5 at its 3′ end. The second probe set, called probe linear (PL), was designed to bind exon 4, which is not a part of this circRNA (Figure 1A). The probes in this set were labeled with Texas Red. This general scheme can be used for any circRNA by choosing the exon(s) included in the circRNA to be the target region for the PC probe set, while the exon(s) only present in linear RNA serve as the target for the PL probe set. The standard protocol of smFISH was followed as described previously [34] and outlined in Figure 1B and Figure S1. The cells were hybridized with both probe sets, and were imaged in both the Texas Red and Cy5 channels. The linear transcript is a target for both probe sets, and thus shows a signal in both of the channels. Because the circRNA has a binding site only for the PC probe set, it exhibits a signal only in the Cy5 channel. The signal from PL was pseudo-colored red, and that from PC was pseudo-colored green, which when merged shows yellow-colored spots for linear RNA and green-colored spots for circRNA (Figure 1C). Therefore, both the linear and circular transcripts could be visualized simultaneously using this approach. We demonstrated circFISH in three different cell lines for the same circRNA. We also quantified the average number of RNA molecules of each isotype in individual cells in all three cell lines (Figure 1D). As expected, we noticed a difference in the overall expression amongst the three different cell lines, with A549 (a lung epithelial cell line) showing the highest expression of both linear and circular ZNF609, followed by DLD-1 cells (colon epithelial), while the bone cell line HS5 (bone fibroblast) showed the lowest expression. CircFISH can also determine the distribution patterns of circRNAs and linear RNAs simultaneously. Because it has been reported that some circRNAs localize and function differently than their linear counterparts [18], we did notice a variation in the distribution of these isoforms. In particular, linear ZNF609 was predominantly cytoplasmic (75%), whereas circZNF609 was found to be equally distributed between nuclear and cytoplasmic regions, and this distribution varied amongst different cell lines (Figure 1E). Because the function of circRNAs is largely determined by their cellular localization, this level of resolution is useful for the functional characterization of circRNAs. These results showed that circFISH can be used to identify the tissue-specific expression and differential distribution of circRNAs in individual cells simultaneously, along with their linear isoforms.

### 3.2. Validation of the CircFISH Signal

**Figure 2:**
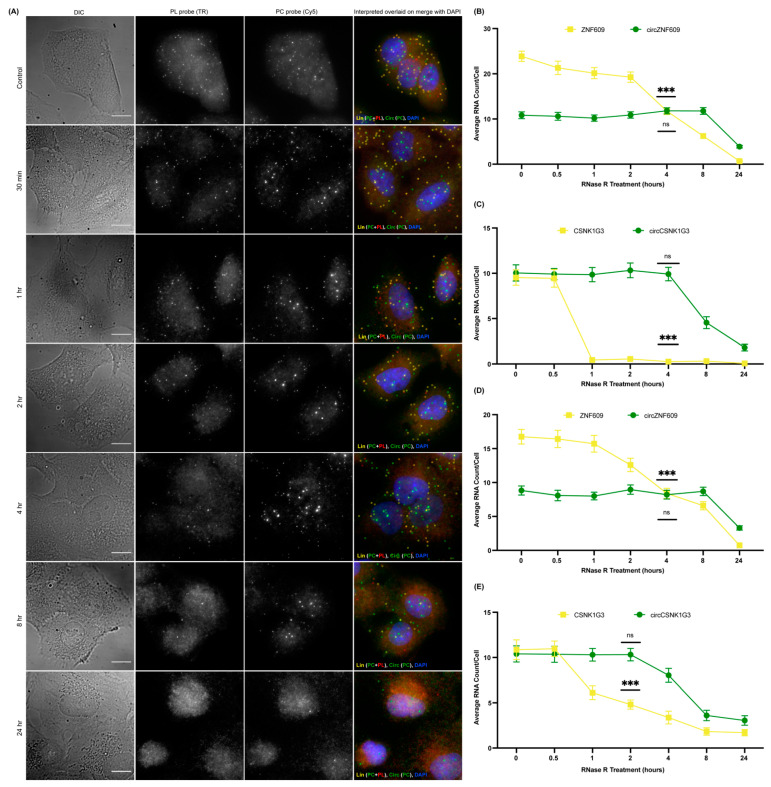
Optimization of *in situ* RNase R treatment for circFISH. (**A**) Representative image panel of A549 cells after 0 min, 30 min, 1 h, 2 h, 4 h, 8 h, and 24 h treatment (from top to bottom) with RNase R before hybridization with PL and PC ZNF609 probes. Columns from the left: DIC; raw merged z stacks of cells for PL probes labeled with TR; raw merged z stacks of cells for PC probes labeled with Cy5; and a merged image of the two channels with TR spots pseudo-colored red and Cy5 pseudo-colored green, overlaid on DAPI, with MATLAB-interpreted spots showing linear RNA as yellow and circular RNA as green. (**B**–**E**) Quantification of the average ZNF609, circZNF609, CSNK1G3, and circCSNK1G3 RNA molecules per cell after MATLAB analysis at each RNase R timepoint in the A549 (**B**,**C**) and DLD-1 (**D**,**E**) cells, respectively. The error bars indicate the 95% confidence interval for at least 100 cells. *** Indicates a significant difference between 0 mins and the particular timepoint, with a *p*-value < 0.001, and ns indicates no significant difference. The scale bar is 5 μm.

**Figure 3:**
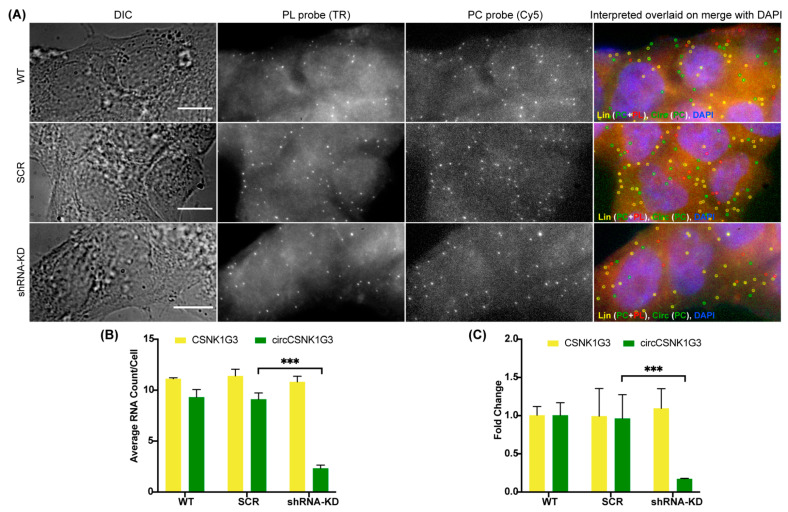
CircFISH after shRNA-mediated knockdown. (**A**) Representative image panel of wild-type (WT) DLD-1 cells (top), DLD-1 cells transduced with scrambled shRNA (SCR) (middle) or with shRNA targeting circCSNK1G3 (shRNA-KD) obtained using PL and PC probes for CSNK1G3. Columns from the left: DIC; raw merged z stacks of cells for PL probes labeled with TR; raw merged z stacks of cells for PC probes labeled with Cy5; a merged image of the two channels with TR spots pseudo-colored red and Cy5 pseudo-colored green, overlaid on DAPI, with MATLAB-interpreted spots showing the linear and circular RNAs as yellow and green spots, respectively. (**B**) Quantification of the CSNK1G3 and circCSNK1G3 signals after MATLAB analysis. The columns represent the average CSNK1G3 and circCSNK1G3 RNA molecules per DLD-1 cell, as analyzed in MATLAB. The error bars indicate the 95% confidence interval for at least 100 cells. (**C**) qRT-PCR data showing the fold change of CSNK1G3 and circCSNK1G3, normalized to Actin mRNA in DLD-1 -WT, SCR and shRNA-KD. The error bars indicate the standard deviation between triplicates. *** Indicates a significant difference with a *p*-value < 0.001. The scale bar is 5 μm.

In order to confirm that the spots obtained by the PC probe set alone are indeed circRNAs and not just degraded or broken fragments of linear RNA, we incorporated an RNase R treatment in our circFISH assay. RNase R is a 5′–3′ exoribonuclease that digests only linear RNA but not circular RNAs, which are resistant to this enzyme [23,37,38]. There are several reports on the use of RNase R on isolated RNA, but its use in fixed cells and in situ has not been extensively reported [28]. Prolonged exposures to RNase R can also degrade circRNAs due to the formation of spontaneous nicks in the circRNAs over time, and due to the inherently unstable nature of the RNAs [39]. Therefore, the incubation time and reaction conditions need to be optimized for in situ RNase R treatment [40]. For this purpose, we treated the fixed and permeabilized cells with RNase R solution prepared in nuclease-free RNase R buffer. We incubated the cells for different periods of time, and performed circFISH after each time interval. We imaged two different pairs of linear and circRNAs, ZNF609 and CSNK1G3 (casein kinase 1 gamma 3), in two different cell lines: A549 and DLD-1. The CSNK1G3 gene has 13 exons; exons 2, 3, and 4 undergo back-splicing as circCSNK1G3, which is reported to play antagonistic roles to that of the product of linear CSNK1G3 RNA (Figure S2A) [41,42]. Therefore, we designed PL to bind to exons 6 to 12, and a PC probe set to bind to exons 2 to 4 of CSNK1G3. CircFISH was able to accurately visualize and measure the effect of RNase R treatment compared to untreated cells (control) (Figure 2A). We found that different cell lines, as well as different RNAs in the same cell line, vary in incubation time from 1 to 4 h to remove most of the linear RNAs while retaining circRNAs (supplementary Figures S2B, S3 and S4). However, upon statistical analysis, we concluded that cells can be treated with RNase R for up to 4 h, leading to a minimal change in circRNA and a significant loss of linear RNA (Figure 2B) for both circRNAs in multiple cell lines (Figure 2B–E). These results prove the specificity of circFISH by validating that the green spots seen in Figure 1 indeed result from the circRNAs. Furthermore, we found that in situ RNase R treatment can also be performed after hybridization with probes, indicating that probe binding does not preclude the activity of RNase R on either linear or circular isoforms (supplementary Figure S5). This is due to the presence of the intrinsic ATP-dependent helicase activity of RNase R, which can degrade duplexes with 3′ overhangs [43]. Additionally, we further validated circFISH by specifically reducing the expression of circRNAs using shRNA targeting their BSJ. This allows for the selective reduction of circRNA while the linear RNA levels remain unchanged [18]. We sought to determine whether circFISH will be able to accurately quantify the decrease in the circRNA levels. For this purpose, the DLD-1 cells were transduced with a lentivirus construct expressing shRNA against circCSNK1G3 BSJ. The cells stably expressing the shRNA construct were selected and hybridized with both PC and PL probe sets for CSNK1G3. Control DLD-1 cells expressing a scrambled version of shRNA were also selected. As expected, circFISH was able visualize and quantify the loss of the circRNA signal in the cells expressing shRNA, while no change was observed in the levels of circRNA in the no-treatment control or the cells expressing the scrambled construct (Figure 3A,B). The knockdown of circRNA was also validated by qRT-PCR analysis (Figure 3C). These results further confirm the specificity of the circFISH method.

### 3.3. Simultaneous Imaging of Multiple CircRNAs

**Figure 4:**
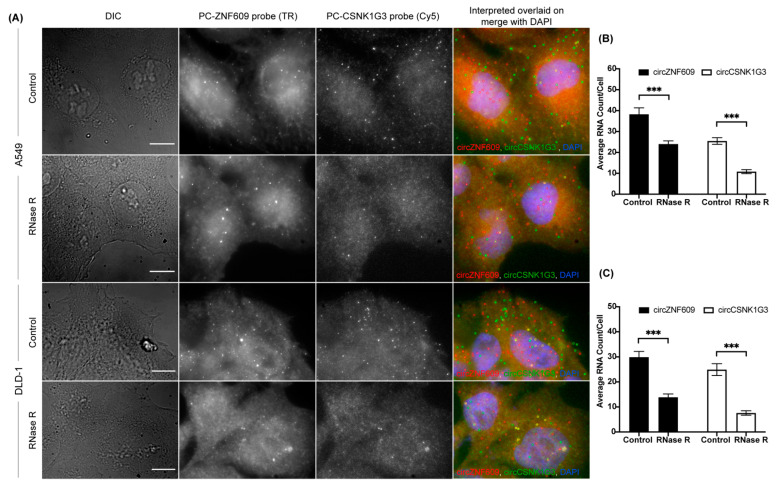
Multiplexing circFISH for the simultaneous visualization of multiple circRNAs using in-situ RNase R treatment. (**A**) A representative image panel of A549 and DLD-1 cells with no (labeled as Control) or 4 h of RNase R treatment (labeled as RNase R), with PC probes of ZNF609 and CSNK1G3. Columns from the left: DIC; raw merged z stacks of cells for the PC ZNF609 probes labeled with TR; raw merged z stacks of cells for the PC CSNK1G3 probes labeled with Cy5; and a merged image of the two channels with the TR spots colored red and Cy5 colored green, overlaid on DAPI with MATLAB-interpreted spots showing circZNF609 as red and circCSNK1G3 as green. (**B**,**C**) Quantification of circZNF609 and circCSNK1G3 signals in the control, and RNase R-treated A549 and DLD -1 cells, respectively. The error bars indicate the 95% confidence interval for at least 100 cells. *** Indicates a significant difference with a *p*-value < 0.001. The scale bar is 5 μm.

The optimization of in situ RNase R treatment in the circFISH assay also enables us to multiplex the circFISH assay. In order to demonstrate this, we treated the cells with RNase R for 4 h and then hybridized them using multiple PC probe sets, each of which were specific for a different circRNA and coupled with a distinct fluorophore. We used PC probe sets for circZNF609 coupled with Texas red and for circCSNK1G3 coupled with Cy5 together to hybridize A549 and DLD-1 cells with or without RNase R treatment (Figure 4A). In the case of untreated cells, the PC probe set should bind to both linear and circular RNAs. This was indeed the case, as we obtained a good consensus between the total RNAs identified by the PC probe set and the sum total of the linear and circRNAs, as shown in Figure 1. Next, the treatment of RNase R for 4 h eliminated the linear RNA, and we could identify both circRNAs together (Figure 4B,C). Thus, using the optimized RNase R treatment, we could reliably image multiple circRNAs simultaneously for their distribution and expression in individual cells. This multiplexing is limited only by the number of fluorophores that can be distinguished spectrally in the given imaging system.

### 3.4. Combining CircFISH with Other Cellular Staining

**Figure 5:**
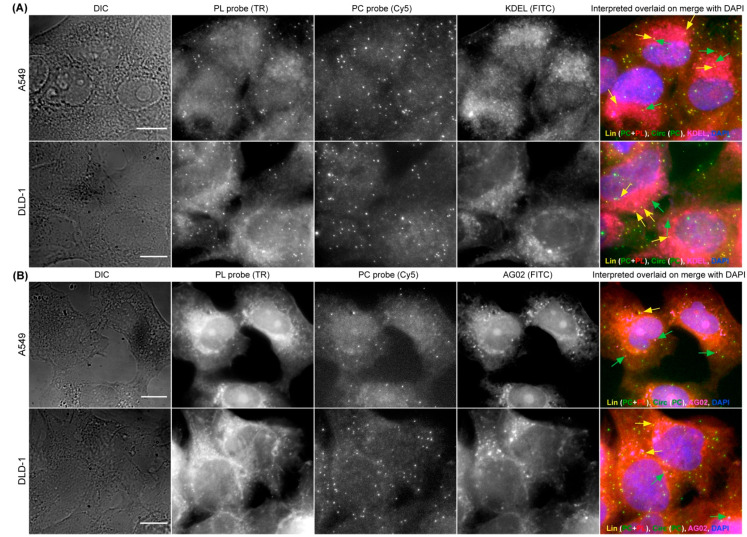
CircFISH in combination with immunofluorescence. (**A**) Representative image panel of A549 and DLD-1 cells treated with PL and PC probes for circFISH of ZNF609, as well as with antibodies against KDEL for ER staining. From the left: DIC; raw merged z stacks of cells for PL ZNF609 probes labeled with TR; raw merged z stacks of cells for PC ZNF609 probes labeled with Cy5; raw merged z stacks of cells for KDEL antibodies tagged with AF488; and a merged image of the three channels with the TR spots colored red, Cy5 colored green and AF488 colored pink, overlaid on DAPI with arrows showing circZNF609 (green) and ZNF609 (yellow) co-localizing with KDEL (pink). (**B**) Representative image panel of A549 and DLD-1 cells treated with PL and PC probes for the circFISH of CSNK1G3, as well as with antibodies against AGO2. Columns from the left: DIC; raw merged z stacks of cells for PL CSNK1G3 probes labeled with TR; raw merged z stacks of cells for PC CSNK1G3 probes labeled with Cy5; raw merged z stacks of cells for AGO2 antibodies tagged with secondary FITC; and a merged image of the three channels with TR spots colored red, Cy5 colored green and FITC colored pink, overlaid on DAPI with arrows showing circCSNK1G3 (green) and CSNK1G3 (yellow) co-localizing with AGO2 (pink). The scale bar is 5 μm.

As the functions of circRNAs are only beginning to be characterized, it is becoming pivotal to acquire an imaging method that can be combined with other cellular staining—such as nucleic acid staining and immunofluorescence (IFA)—so that the circRNAs’ interactions with proteins and other nucleic acids can be visualized and validated. We previously optimized smFISH in combination with IFA and nucleic acid staining in various biological systems [44]. Utilizing DAPI staining during the mounting of circFISH samples, we can clearly define the nuclear and cytoplasmic distribution of circRNAs, as shown in Figure 1C. Furthermore, for the testing of the compatibility of circFISH with IFA, we used counterstains for cellular organelles and proteins in combination with circFISH. First, we used an antibody for KDEL (a marker for endoplasmic reticulum, ER) to label ER along with circFISH probe pairs (PL and PC) for ZNF609, and observed the colocalization of KDEL and linear/circular ZNF609 (Figure 5A). In order to further demonstrate the compatibility of circFISH with IFA, we used an antibody for the argonaute protein (AGO2) along with the circFISH probe pairs for CSNK1G3 RNA. We processed these images to identify the colocalization of AGO2 with linear and circular CSNK1G3 RNA, which is indicated by yellow and green arrows, respectively, in the last panel of Figure 5B. In order to demonstrate the flexibility of circFISH, we performed circFISH and IFA in alternative orders. In the first iteration, we first performed ZNF609 RNA circFISH as described above. After washing off the unbound probes, we conducted IFA using a KDEL biomarker for ER staining. Reversing the order in the second iteration, where IFA was followed by circFISH, yielded similar results (Supplementary Figure S6). These results confirmed that circFISH can be combined seamlessly with other cellular staining procedures, and hence is a useful tool for the functional characterization of circRNAs. 

### 3.5. Optimization of CircFISH for Multiple Sample Types

**Figure 6:**
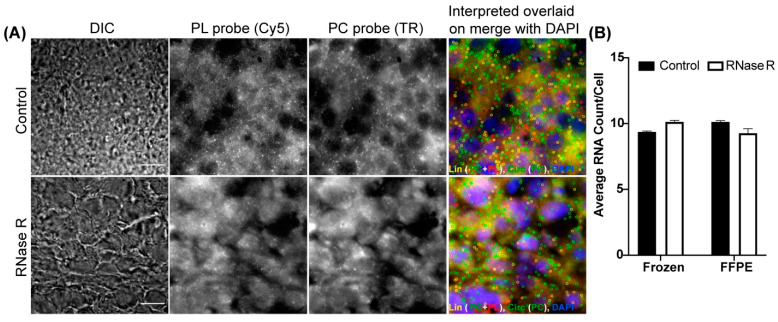
Visualizing circPOK in frozen and FFPE mouse tissues using circFISH. (**A**) Representative image panel of frozen mouse melanoma tissue with (labeled as RNase R) or without (labeled as Control) 2 h RNase R treatment before the PL and PC probe hybridization. Columns from the left: DIC; raw merged z stacks of cells for PL probes labeled with Cy5; raw merged z stacks of cells for PC probes labeled with TR; and a merged image of the two channels with TR spots pseudo-colored green and Cy5 pseudo-colored red, overlaid on DAPI with MATLAB-interpreted spots showing the linear and circular RNAs as yellow or green spots, respectively. (**B**) Quantification of the circPOK RNA per cell in frozen and FFPE mouse tissue samples between the control and RNase R-treated samples. The error bars indicate the 95% confidence interval. At least 100 cells were counted for each condition. The scale bar is 5 μm.

We utilized adherent cultured cells to demonstrate the wider applicability of circFISH. Because most of the clinical samples are either frozen or formalin-fixed paraffin-embedded (FFPE) tissue sections, we decided to optimize circFISH in these clinical sample types. For this purpose, we utilized fresh-frozen sections and FFPE sections from a mouse xenograft of melanoma to detect circPOK, a circular RNA produced from the ZBTB7a gene (also known as leukemia release factor, LRF). This circRNA was characterized by our group in collaboration with Dr. Pandolfi’s lab, where we utilized circFISH to analyze the distribution and function of circPOK in both human and mouse cell lines [18]. In this case, exon 2 circularizes to form circPOK, so we designed a PC probe set for exon 2 and a PL probe set for exon 3 of the transcript (supplementary Figure S7A). As expected, we were able to identify both linear and circular isoforms distinctly in both the fresh-frozen sections (Figure 6A) and FFPE sections (supplementary Figure S7B). We also performed RNase R treatment and confirmed the identity of the circRNA signal in these sections (Figure 6A,B). This shows the ability of circFISH to be used in clinical samples.

### 3.6. Expression Profiling of CircRNAs Using CircFISH

**Figure 7:**
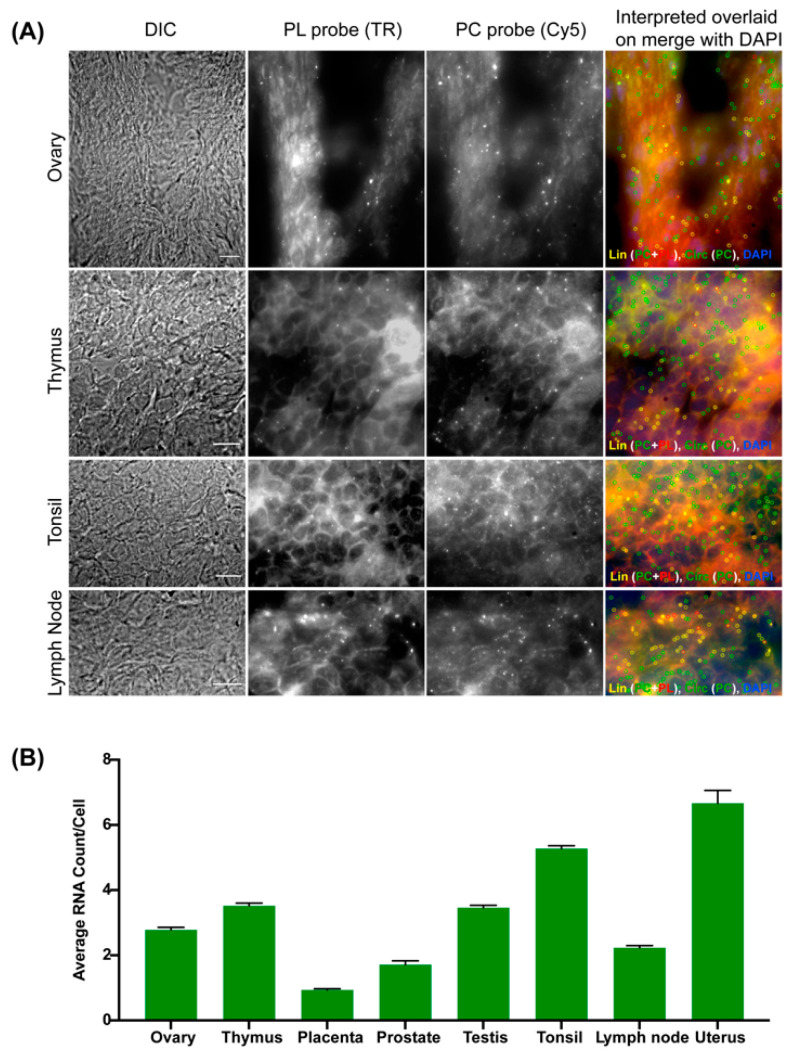
Expression profiling of circPOK in frozen human tissue samples using circFISH. (**A**) Representative image of the circFISH imaging of a panel of the frozen human tissue array (containing ovary, thymus, tonsil, and lymph node) with PL and PC probes. Columns from the left: DIC; raw merged z stacks of cells for PL probes labeled with TR; raw merged z stacks of cells for PC probes labeled with Cy5; and a merged image of the two channels with TR spots pseudo-colored red and Cy5 pseudo-colored green, overlaid on DAPI with MATLAB-interpreted spots showing the linear and circular RNAs as yellow and green, respectively. (**B**) Quantification of the circPOK signals to identify the average circPOK RNA count per cell across multiple tissue types. The error bars indicate the 95% confidence interval. At least 100 cells were counted for each condition. The scale bar is 5 μm.

In order to develop an expression profile for circRNAs across multiple tissues, we used a frozen tissue array (Amsbio, Abingdon, UK, T6234433) with eight human tissues fixed on a slide [5,45]. We hybridized with a circPOK probe pair to verify whether circFISH can detect the endogenous circPOK expression levels in different tissues. We were able to detect distinct signals for circPOK RNAs in these tissues (Figure 7A,B). We noticed a differential expression of circPOK which correlates well with tissue expression atlas databases. Due to the single-molecule level sensitivity of circFISH, we were able to detect the expression of circPOK in tissues for which tissue expression database reports were lacking [25,46,47]. These findings confirm the accuracy and sensitivity of circFISH in providing contextual imaging of circRNAs in different sample types. 

## 4. Discussion

With the recent acknowledgement of the role of circRNAs as biomarkers, a robust and sensitive method for the imaging of their cellular distribution is required. The contextual and spatial distribution of circRNAs must be ascertained in order to accurately determine their function in the cell [48]. For example, a recent imaging-based analysis identified that the most well-studied oncogenic circRNA, CDR1as (ciRS-7), which was earlier believed to be expressed by colon cancer cells, is actually expressed by stromal cells in the tumor microenvironment [49,50]. However, because the existing imaging-based approaches for circRNA detection rely solely on a single unique BSJ in the circRNA, these approaches are often plagued with non-specific binding and high background noise. Such background noise often leads to false-positive signals, hindering their use in clinical applications. Furthermore, most of the current methods have very limited multiplexing capabilities. Our work has addressed this unmet need by providing circFISH as a simple and sensitive imaging-based method for the specific detection of circRNAs in fixed cells at a single-molecule resolution. CircFISH is based on smFISH, which is the ‘gold standard’ method for the single-molecule resolution imaging of RNAs in individual cells (Figure 1). The incorporation of an optimized RNase R treatment in situ allowed us to validate the circFISH signal with high confidence. This also enabled us to multiplex circFISH for the simultaneous imaging of multiple circRNAs (Figure 4).

A few protocols have been developed to optimize the RNase R treatment for isolated RNA for sequencing purposes. However, to the best of our knowledge, this is the first report on the time-course optimization of in situ RNase R treatment for the imaging and validation of circRNAs [51]. Our findings suggest that 4 h of RNase R treatment is sufficient to degrade most of the linear RNAs, while circRNAs remain unaffected (Figure 2). However, prolonged digestions with RNase R can start to degrade circRNAs over time. We also observed that the optimal RNase R incubation time varies between cell lines, as well as between different RNAs. Therefore, we recommend that a time-course optimization of RNase R treatment be performed in case there is a minimal loss of linear RNA and/or a significant loss of circRNA with the 4 h RNase R treatment. We further demonstrated the use of this assay in combination with other imaging assays, which is particularly useful for the functional characterization of circRNAs. In addition to our previous demonstration of the use of circFISH in the analysis of the interaction between circPOK and ILF 2/3 protein [18], we demonstrated the colocalization of circRNAs with argonaute protein, as well as with an ER marker (Figure 5). In this context, the ER colocalization of circZNF609 is interesting, as it is one of the few circRNAs which are reported to have a coding potential [36,52]. Therefore, the colocalization of circZNF609 with ER further suggests that circZNF609 is likely to be coding for a peptide. Finally, we showcased the utility of circFISH in clinical samples, such as fresh-frozen and FFPE samples. These results make circFISH a useful assay for use in the analysis of clinical samples (Figure 6). Here, we suggest the use of a strict RNase-free environment to avoid the degradation of linear RNAs from FFPE sections. We also noticed that fresh-frozen sections provide cleaner signals compared to FFPE sections with the PL probe set for linear RNAs. CircRNAs, however, are more stable and resistant to exonucleases, as evidenced by the clear and distinct signals obtained using the PC probe pair for both fresh-frozen and FFPE sections. This further attests that circRNAs, which are very promising targets in clinical samples, can be imaged reliably using circFISH [53]. We detected endogenous levels of circPOK in tissues that are not reported in the tissue expression database to express circPOK (Figure 7). This could be explained by the fact that these expression databases are built using high-throughput sequencing data and do not include single-cell data, and are therefore likely to miss very low levels of cellular RNA expression. Alternatively, a conceivable presence of broken fragments of linear RNA in our samples could allow the binding of the PC probes (as the tissue array slide was not treated with RNase R prior to the imaging). However, the fact that our analysis in the cell lines consistently shows the high correlation of the number of circRNA spots obtained before and after RNase R treatment provides proof that such signals are not confounding factors for the accuracy of analysis. Furthermore, our observed similarities of circRNA expression patterns with those reported in public expression databases supports the specificity and reliability of circFISH.

CircFISH has the same inherent limitations as those of Fusion FISH (33). One major limitation is the size of the RNA target. Because the signal generated from a single probe (containing just one fluorophore) is not strong enough to be visualized, a set of at least 15–20 probes is required to obtain a discrete diffraction-limited spot. Each probe needs to be 18–20 nt long, and a 2 bp is needed in between the binding sites of each probe in order to avoid quenching by the adjacent probe. Therefore, a minimum length of 300–400 bp of the target RNA is required. Furthermore, although regular fluorescence and/or a confocal microscope can be used, a good-quality cooled-CCD camera must be used, as it helps to retain most of the signal and provides more clear images with the lowest number of probes.

In future, circFISH could be combined with flow cytometry to enable a high throughput characterization of circRNAs in clinical samples. CircFISH could be used to elucidate the mechanism of action of circRNAs by utilizing PC probe sets in RNA probe-based affinity methods like Chromatin Isolation by RNA Purification (ChIRP) or RNA Affinity Purification (RAP) [54]. In these methods, the probe set can be labeled with biotin instead of fluorophores. The cell lysates are treated with RNase R to deplete linear RNAs and then hybridized with the biotin-labeled probes. These probes will bind the target circRNAs, and will pull down RNA, DNA and proteins interacting with the target circRNAs [48]. We previously demonstrated the use of circFISH for the pulldown of the interacting protein partners of circPOK [18]. Once the interacting partners are identified, this helps to identify and/or alter circRNA function, and to develop specific diagnostic and targeted therapeutic approaches [19,45,55].

## 5. Conclusions

We present circFISH as a versatile circRNA imaging method, which is more economical and less tedious compared to other methods for the imaging of circRNAs. The protocol is similar to smFISH with a single-step hybridization with probes. The only additional step is the RNase R treatment in the case of validation and multiplexing. The analysis is very straightforward, and simply requires the identification of distinct spots and the colocalization of the spots between two channels. This can be performed with any image analysis package, such as freely available ImageJ software. Finally, like smFISH, circFISH offers sensitivity and specificity comparable to quantitative real-time PCR, making it the most versatile tool for the accurate, sensitive and reliable imaging of circular and linear RNA isoforms in individual cells at a single-molecule resolution.

## Data Availability

The data are contained within the article and Supplementary Materials. The custom algorithms used for the image processing and the plasmids generated in the study are available upon request.

## Supplementary Materials

The following are available online at https://www.mdpi.com/article/10.3390/cancers14020428/s1. Figure S1: A detailed work flow of the circFISH assay; Figure S2: In-situ RNase R treatment time course for the validation of circCSNK1G3 in A549 cells; Figure S3: In-situ RNase R treatment time course for the validation of the circZNF609 signal in DLD-1 cells; Figure S4: In-situ RNase R treatment time course for the validation of circCSNK1G3 in DLD-1 cells; Figure S5: Quantification of in-situ RNase R treatment after circFISH hybridization for the validation of circZNF609; Figure S6: CircFISH in combination with other cellular staining; Figure S7: Visualization of circPOK in FFPE mouse tissues using circFISH. Table S1: List of primers and probes used in the study.
