# Supplementary material for: CircFISH: A Novel Method for the Simultaneous Imaging of Linear and Circular RNAs
Source: Cancers (Basel). 2022 Jan 15;14(2):428. doi: 10.3390/cancers14020428 (PMC8773908; doi:10.3390/cancers14020428)
Supplement: Supplementary file 1 [file cancers-14-00428-s001.zip › cancers-1527107-supplementary/Supplementary files/circFISH_Supplementary Figures S1-S7 FINAL.pdf]

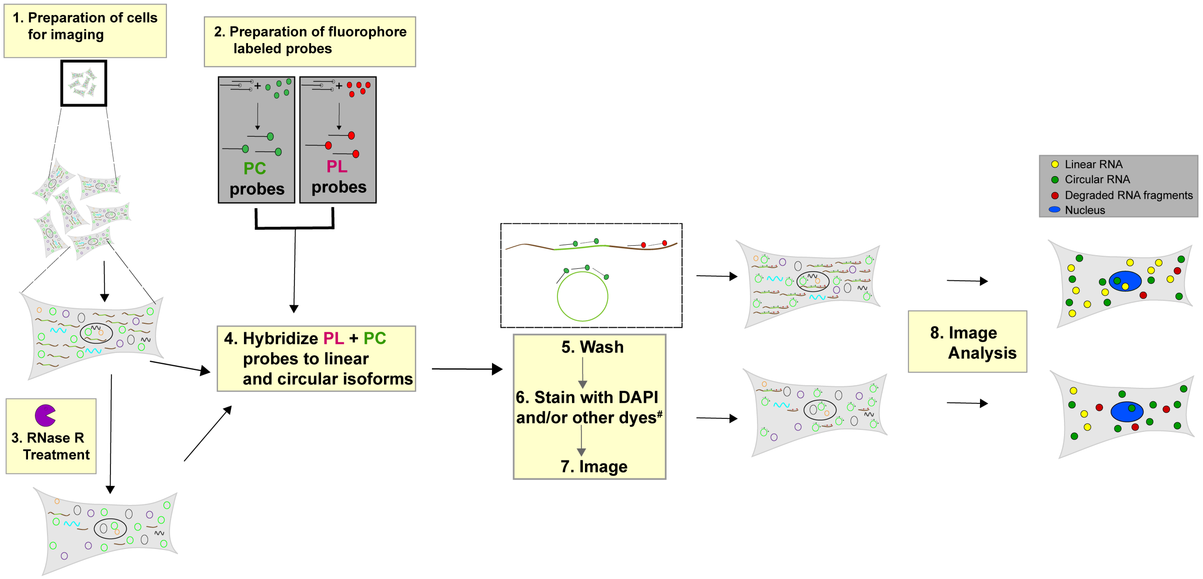

Figure S1: Workflow for circFISH: (1) Cells are grown on glass cover slips that have been precoated with 0.1% gelatin to ~90% confluency. Cells are fixed with 4% formaldehyde for 10 minutes and then permeabilized by adding 70% ethanol and incubated at 4°C for at least 30 minutes. Permeabilized cells can be kept at 4°C for months until ready to hybridize. **(2)** The target RNA sequences are identified using Ensembl or UCSC genome browsers and CircInteractome. The custom Stellaris RNA FISH probes with 3′ amino group modification are designed using Stellaris probe designer tool available on https://www.biosearchtech.com/support/tools/design-software/stellaris-probe-designer). The probes are then pooled and coupled *en mass* with fluorescence dyes. Dye-coupled probes are purified from unlabeled probes and the loose dye using reverse phase C18 columns on HPLC. The labeled probes can be stored at -20°C until ready to use. **(3)** An optional step of RNase R treatment can be performed to at room temperature to deplete linear RNAs. **(4)** The cells are hybridized with the probes and incubated at 37°C overnight in a water bath. **(5)** The cells are washed for 15 minutes for a total of 4 times to remove any unbound probes. **(6)** Cells are stained with DAPI, and **(7)** mounted using deoxygenated mounting medium and imaged using 100X oil objective in a fluorescence microscope equipped with cooled CCD camera. **(8)** The images are then merged and analyzed with Fiji and MATLAB. The # symbol at step 6 indicates the step where other counter-staining procedures (for cellular organelles or immunofluorescence) can be performed.

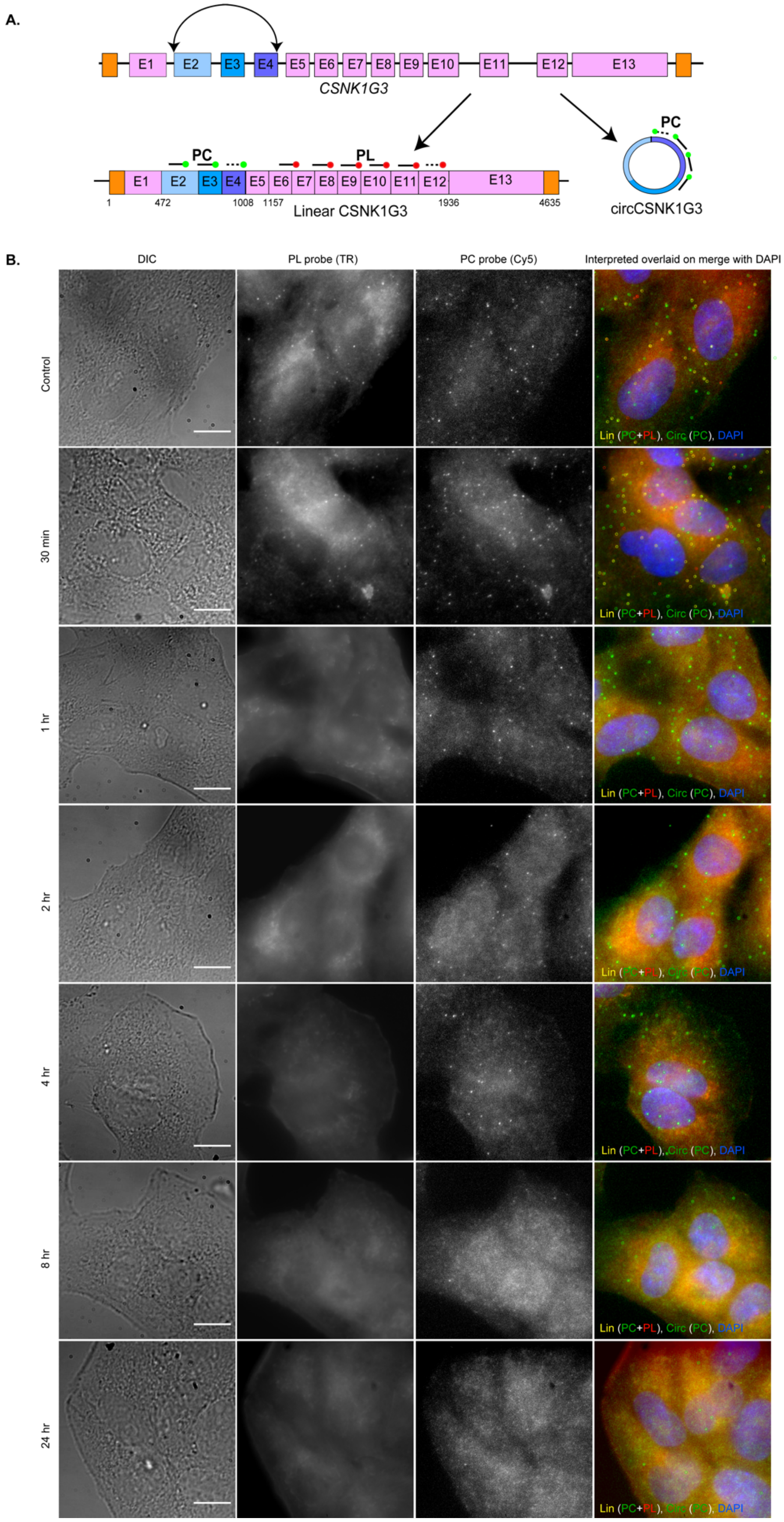
A.
E1
E2
E3
E4
E5
E6
E7
E8
E9
E10
E11
E12
E13
CSNK1G3
PC
PL
1
472
1008
1157
1936
4635
Linear CSNK1G3
circCSNK1G3
B.
DIC
PL probe (TR)
PC probe (Cy5)
Interpreted overlaid on merge with DAPI
Control
30 min
1 hr
2 hr
4 hr
8 hr
24 hr
Lin (PC+PL), Circ (PC), DAPI

Supplementary Figure S2: Optimization of *in situ* RNase R treatment for circFISH for circCSNK1G3 in A549 cells. (A) Production of the linear and circCSNK1G3 RNA isoforms of CSNK1G3 gene is illustrated. The PL and PC probes are represented with straight lines ending with red and green filled circles, respectively. **(B)** A representative image panel of A549 cells hybridized with PL and PC CSNK1G3 probes after 0 mins, 30 mins, 1 hour, 2 hours, 4 hours, 8 hours, and 24 hours of RNase R treatment (from top to bottom). From left: DIC, raw merged z stacks of cells for PL probes labeled with TR, raw merged z stacks of cells for PC probes labeled with Cy5, a merged image of the two channels with TR spots pseudo-colored as red and Cy5 pseudo-colored as green, overlaid on DAPI with MATLAB interpreted spots showing linear RNA as yellow and circular RNA as green circles. Scale bar is 5 μm.

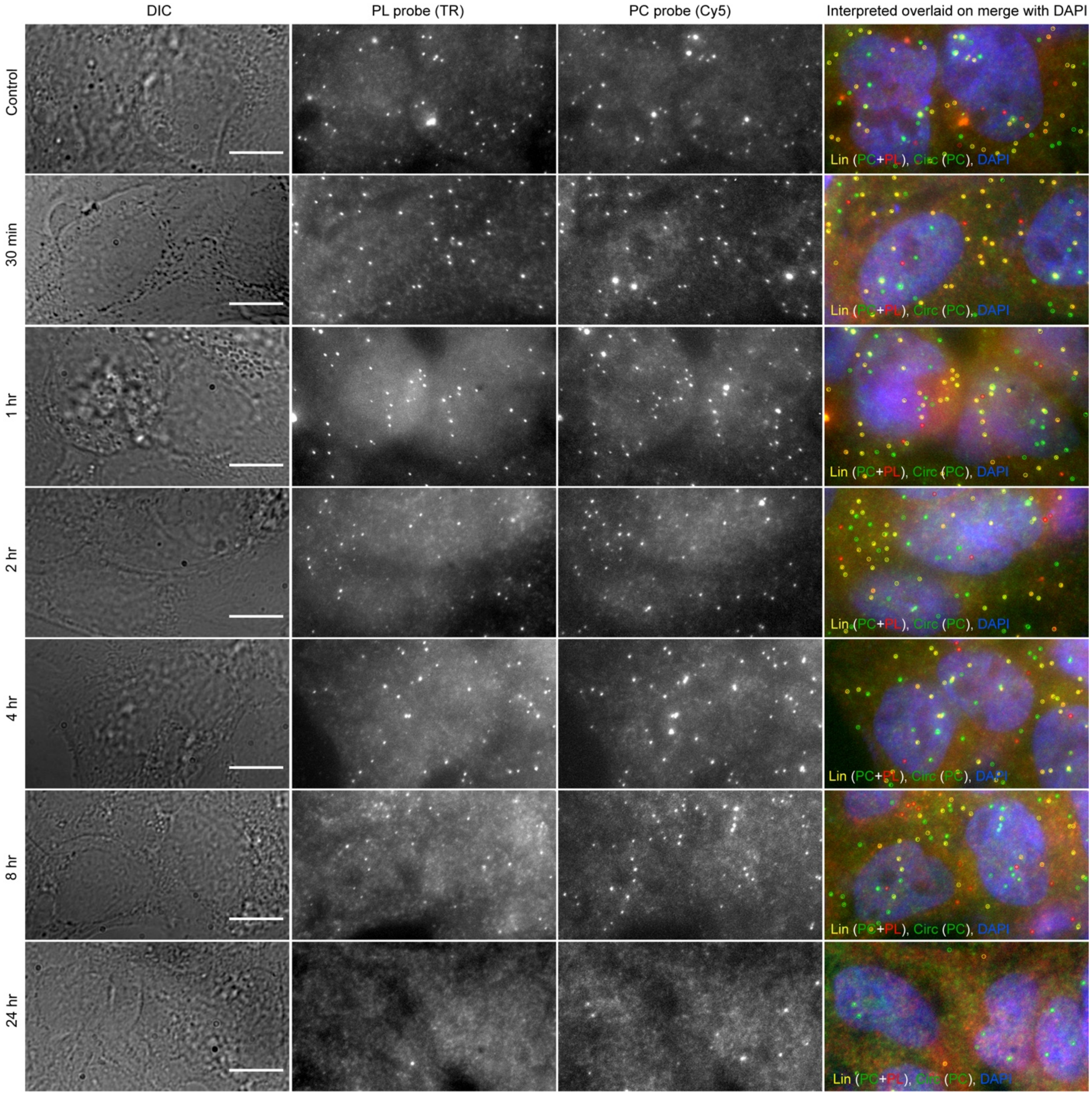

Supplementary Figure S3: Optimization of *in situ* RNase R treatment for circFISH for circZNF609 in DLD-1 cells. A representative image panel of DLD-1 cells hybridized with PL and PC ZNF609 probes after 0 mins, 30 mins, 1 hour, 2 hours, 4 hours, 8 hours, and 24 hours of RNase R treatment (from top to bottom). From left: DIC, raw merged z stacks of cells for PL probes labeled with TR, raw merged z stacks of cells for PC probes labeled with Cy5, a merged image of the two channels with TR spots pseudo-colored as red and Cy5 pseudo-colored as green, overlaid on DAPI with MATLAB interpreted spots showing linear RNA as yellow and circular RNA as green circles. Scale bar is 5 μm.

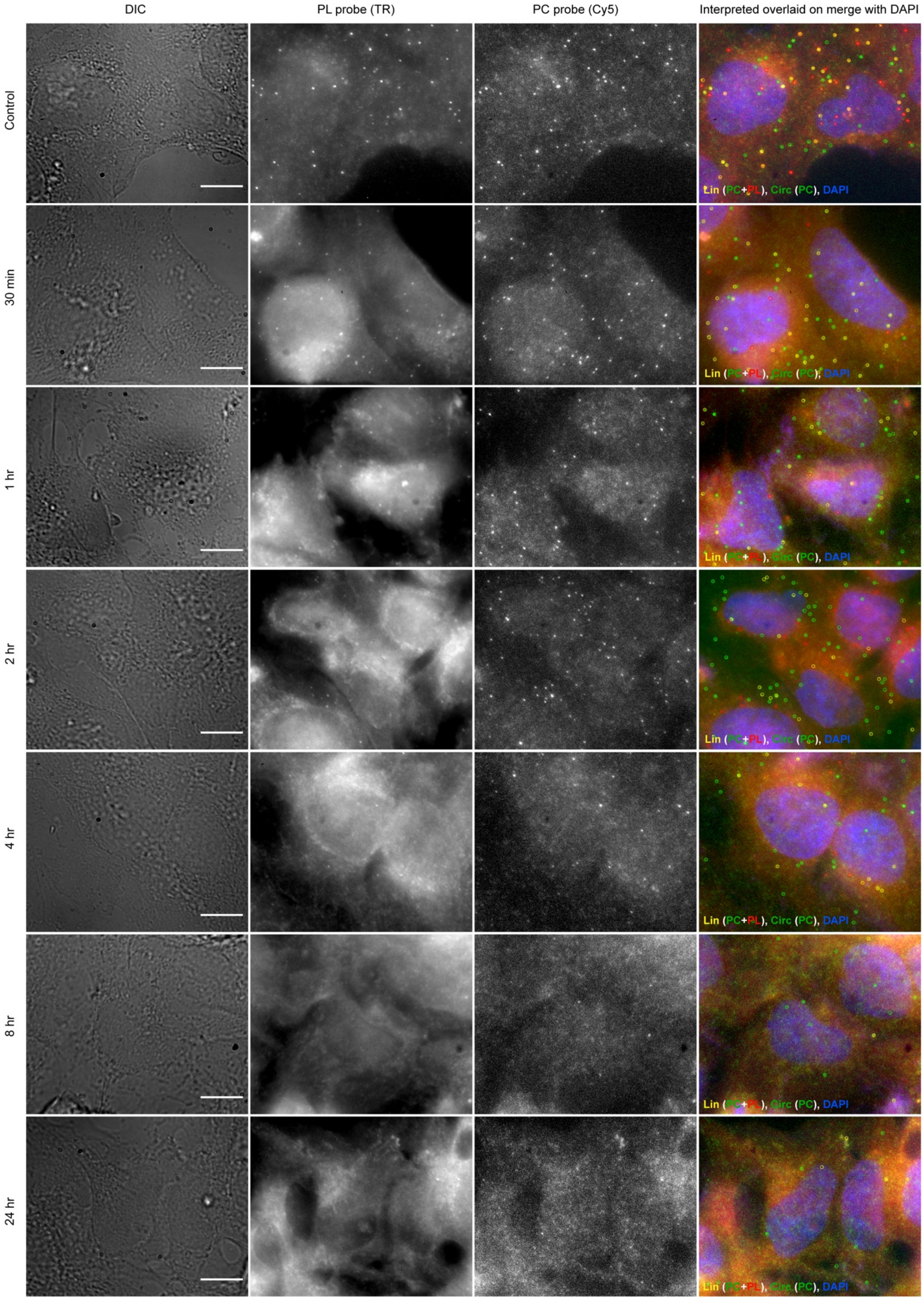

DIC
PL probe (TR)
PC probe (Cy5)
Interpreted overlaid on merge with DAPI
Control
30 min
1 hr
2 hr
4 hr
8 hr
24 hr
Lin (PC+PL), Circ (PC), DAPI

Supplementary Figure S4: Optimization of *in situ* RNase R treatment for circFISH for circCSNK1G3 in DLD-1 cells. A representative image panel of DLD-1 cells hybridized with PL and PC CSNK1G3 probes after 0 mins, 30 mins, 1 hour, 2 hours, 4 hours, 8 hours, and 24 hours of RNase R treatment (From top to bottom). From left: DIC, raw merged z stacks of cells for PL probes labeled with TR, raw merged z stacks of cells for PC probes labeled with Cy5, a merged image of the two channels with TR spots pseudo-colored as red and Cy5 pseudo-colored as green, overlaid on DAPI with MATLAB interpreted spots showing linear RNA as yellow and circular RNA as green. Scale bar is 5 μm.

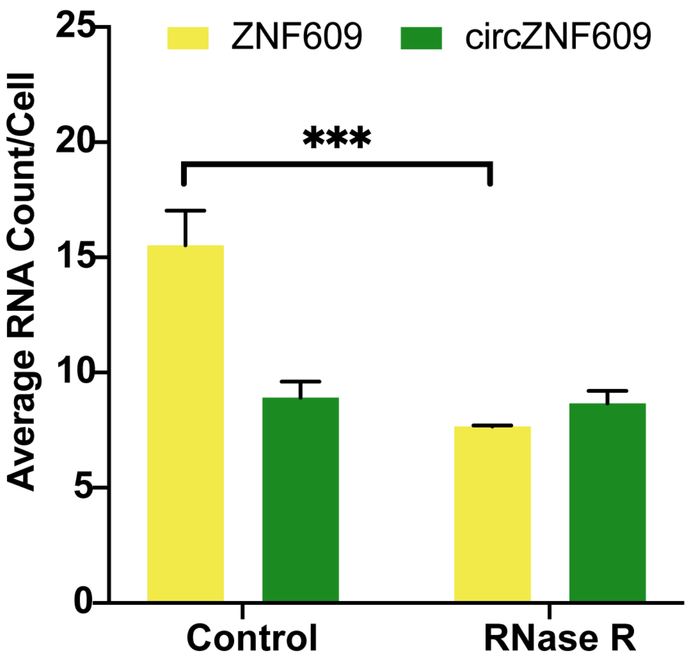

Supplementary Figure S5: Quantification of In-situ RNase R treatment after circFISH hybridization for validating circZNF609. Quantification of the average ZNF609 and circZNF609 RNA molecules per cell with 0 hours (control) or 4 hours (RNase R) of RNase R treatment after MATLAB analysis. Error bars indicate the 95% confidence interval for at least 100 cells. *** indicates significant difference with p-value < 0.001.

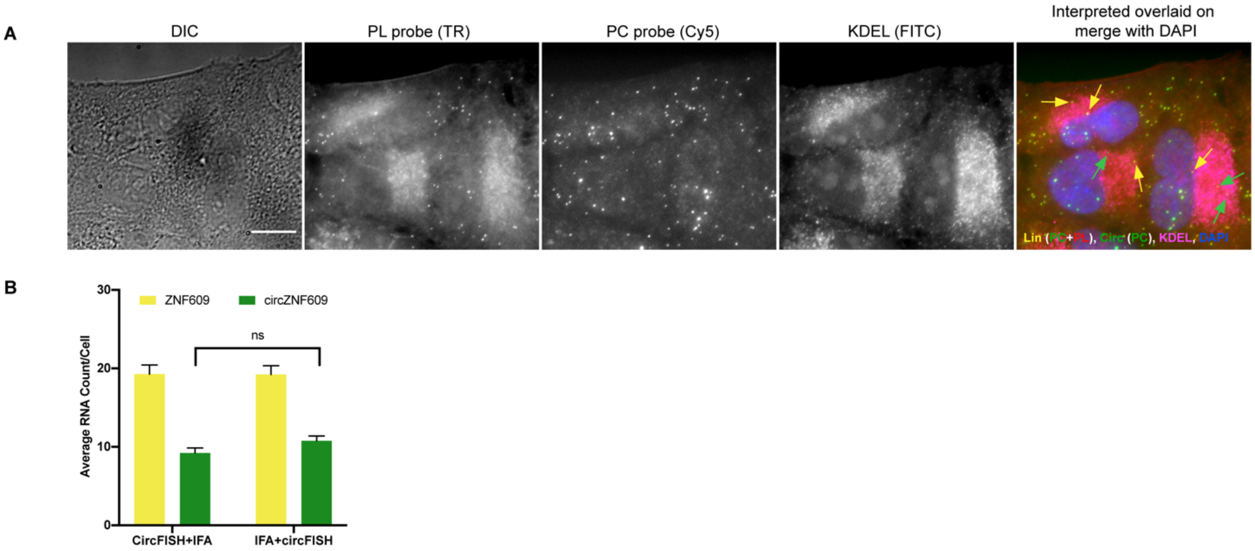

Supplementary Figure S6: CircFISH in combination with other cellular staining. (A) Co-localization of ZNF609 and circZNF609 with KDEL visualized by combining immunofluorescence staining followed by circFISH. A representative image panel of A549 cells with PL and PC probes of ZNF609 and antibodies against KDEL for ER staining. From left: DIC, raw merged z stacks of cells for PC ZNF609 probes labeled with TR, raw merged z stacks of cells for PC ZNF609 probes labeled with Cy5, raw merged z stacks of cells for KDEL antibodies tagged with AF488, a merged image of the three channels with TR spots colored as red, Cy5 colored as green and AF488 colored as pink overlaid on DAPI with arrows showing circZNF609 (green) and ZNF609 (yellow) co-localizing with KDEL (pink) in A549 cells. **(B)** Quantification of the average ZNF609 and circZNF609 RNA molecules per cell after MATLAB analysis between different order of circFISH and immunofluorescence staining protocol. Error bars indicate the 95% confidence interval for at least 100 cells. "ns" indicates no significant difference. Scale bar is 5 μm.

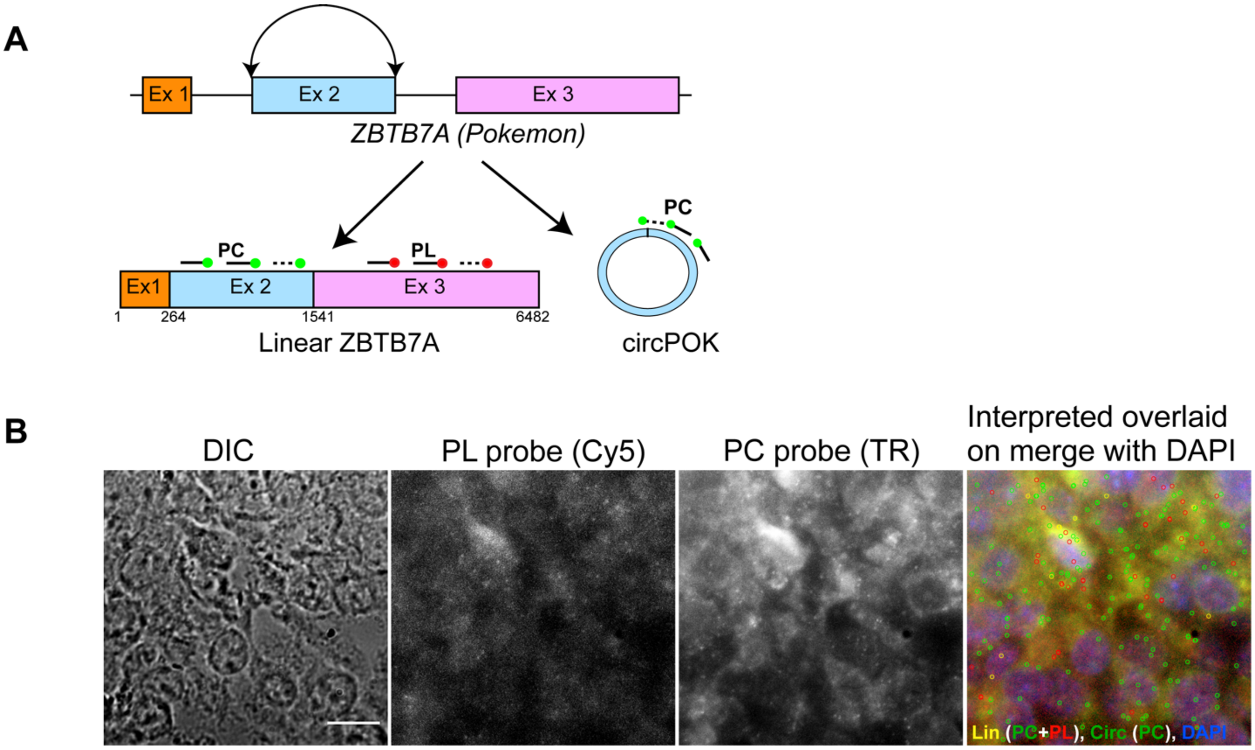

Supplementary Figure S7: Visualizing circPOK in FFPE mouse tissues using circFISH. (**A**) Production of the linear and circPOK RNA isoforms of *ZBTB7A* gene is illustrated. The PL and PC probes are represented with straight lines ending with red and green filled circles, respectively. **(B)** CircFISH assay of circPOK in FFPE samples of mouse xenograft melanoma tissue. A representative image panel of FFPE mouse tissue section hybridized with PL and PC probes after treatment with RNase R for 2 hours. From left: DIC, raw merged z stacks of cells for PL probes labeled with Cy5, raw merged z stacks of cells for PC probes labeled with TR, a merged image of the two channels with TR spots pseudo-colored as green and Cy5 pseudo-colored as red, overlaid on DAPI with MATLAB interpreted spots showing linear RNA as yellow and circular RNA as green circles. Scale bar is 5 μm.
